# Ultrafiltration Membranes System: A Proposal to Remove Emerging Pollutants in Urban Wastewater

**DOI:** 10.3390/membranes12121234

**Published:** 2022-12-07

**Authors:** Ana Belén Lozano Avilés, Francisco Del Cerro Velázquez, Fernando Lozano Rivas

**Affiliations:** 1 Water Department, VECTORIS, S.L., Espinardo, 30100 Murcia, Spain; 2 Department of Electromagnetism and Electronics, Faculty of Chemistry, Campus of Espinardo, 5, Espinardo, 30100 Murcia, Spain

**Keywords:** reuse, membrane biological reactor, nutrients, pathogens, micropollutants, emerging pollutants

## Abstract

Considering the important role that wastewater reuse plays in the water cycle and in the current water scenario immersed in a severe drought, the search for technologies that allow obtaining quality water for reuse is increasingly relevant. In this sense, the membrane biological reactor (MBR) is an alternative to traditional activated sludge systems, in which the separation of biomass and treatment water is carried out by membrane filtration instead of decantation. This study made it possible to confirm the presence of emerging pollutants in the wastewater entering the WWTPs under study, to study the behavior and performance of MBR systems with hollow fiber membranes and flat membranes in obtaining reclaimed wastewater for subsequent reuse, and to compare it with the degree of elimination obtained in conventional biological treatment. It has been demonstrated that this technology is almost 100% effective in the elimination of nutrients, organic matter, pathogens, organic micropollutants, metals, etc., and has achieved different percentages of success in eliminating emerging pollutants depending on their nature: 35% in insecticides and herbicides, 45% in anxiolytics, psychiatric drugs, and industrial disinfectants, 75% in antibiotics, and around 100% in analgesics, anti-inflammatory drugs, and hormones. It has also contributed to the establishment of monitoring protocols for emerging pollutants in the WWTPs under study and to the evaluation of their risks, as well as the development and implementation of advanced regeneration systems that are economically favorable for increasing the quality of WWTP effluents for their reuse.

## 1. Introduction

The use of effluents from wastewater treatment plants (WWTP) is a viable alternative to mitigate water stress in regions where lack of water is a problem in the short to long term. This is the case in the eastern part of Spain, where there is an imbalance between the high demand for freshwater (mainly for agricultural uses) and the scarce water resources available.

Even though in this territory there is an absolute awareness of the exploitation and use of reclaimed water, with reuse figures of over 95% in recent years [1], there is a great lack of knowledge about the long-term environmental consequences of this practice.

Different proposals, strategies, and regulations at international, European, and national levels reflect the need for safe reuse. An example of this is the Sustainable Development Goal on Water (SDG6), promoted by the UN and focusing on integrated water resources management, reflecting the need for safe water reuse worldwide by 2030 [2]. In the same vein, the European Parliament and the Council published the regulation on minimum requirements for water reuse [3]. Among the main objectives of this regulation is to guarantee that reclaimed water is safe for agricultural irrigation, ensure a high level of protection for the environment and human and animal health, promote the circular economy, and contribute to the fulfillment of the European Union’s water policies [4,5,6] by addressing the scarcity of this heritage asset. In Spain, the quality and uses of reclaimed water are regulated by the entry into force of the Royal Decree [7], which establishes the minimum mandatory quality criteria required for the use of reclaimed water according to its uses.

Even though all legislation related to the quality of reclaimed water is increasingly restrictive, reuse is not risk-free. At present, the presence of influents and effluents from WWTPs of the so-called Contaminants of Emerging Concern (CECs) poses a new challenge in terms of the quality of treated water, mainly when it comes to enhancing its reuse options. So much so that, although most CECs are not covered by current regulations, some of them are already included in lists of priority substances in water and are therefore among the priority lines of research of the main bodies dedicated to public and environmental health, such as the World Health Organization (WHO) or the European Commission and are therefore susceptible to future regulations.

In general terms, CECs are defined as previously unknown or unrecognized contaminants whose presence in the environment is not necessarily new, but there is concern about the possible consequences of their presence. They cover a wide variety of everyday products with both industrial and domestic applications (pharmaceutical compounds, detergents, hormones, beauty and personal care products, pesticides, microplastics, etc.). Although CECs are generally found in WWTP influents at low concentrations (in the order of ng/L or μg/L), conventional WWTPs are not designed for the removal of these types of compounds, so the most persistent compounds remain in the effluents.

In this situation, WWTPs are one of the main routes of entry of CECs into the environment. Continued discharge of effluents containing CECs and chronic exposure to CECs can present a potential risk to natural ecosystems. This problem is further aggravated when WWTP effluents are intended to be used for crop irrigation, as these compounds can be absorbed by plants and thus enter the food chain, posing health risks. It is therefore essential to carry out detailed studies on different but complementary issues:

– Establishment of protocols for monitoring CECs and risk assessment, both for health and the environment, with the aim of establishing new quality parameters for safe reuse.
Study of the effectiveness in the elimination of CECs of the treatment technologies installed in WWTPs.
Development and implementation of advanced regeneration systems that are economically favorable in terms of increasing the quality of WWTP effluents for reuse, guaranteeing the elimination of CECs, thus minimizing the associated environmental risks, and enabling the transition towards a more circular model.

For the first issue, the scientific community is required. In this regard, in recent years, many authors [8,9,10] have developed specific analytical methodologies for the presence of certain CECs in environmental matrices, as well as guidelines for risk analysis in different environmental compartments, life cycle studies, etc.

The third issue is based on the development and optimization of new complementary or advanced treatments to improve the removal performance of persistent compounds that cannot be removed in secondary treatments. In this perspective, different technologies based on filtration techniques (nanofiltration, reverse osmosis, etc.), adsorption (powdered activated carbon (PAC) or granular activated carbon (GAC)), or advanced oxidation processes (UV/TiO2, photo-Fenton, etc.) are currently being investigated [11,12,13,14]. However, most of these technologies are not widely used, mainly due to high costs, which makes their implementation in WWTPs difficult. The evolution in the reduction of operation and maintenance costs of membrane technology, mainly due to the development of methodologies for energy efficiency, has increased the implementation of this type of technology [15,16,17].

About the second question, and on which we focus our work, we will refer to the analytical development for monitoring and control of a list of the most frequent emerging pollutants in wastewater arriving at the WWTP and effluent outflow to check the effectiveness of the treatment technologies installed in the WWTPs. Specifically, the behavior and performance of flat membrane and hollow fiber systems in obtaining reclaimed wastewater for subsequent reuse are studied.

To this end, a characterization of the inlet and permeate water was carried out in terms of the usual parameters in purification control (chemical oxygen demand, nitrogen, phosphorus, pathogens, etc.) and specific micropollutants (pharmacological principles, etc.). The aim of the project was to study the elimination of different types of pollutants (nutrients, organic matter, pathogens, organic micropollutants, metals, etc.) in each of the WWTPs, comparing the elimination performance of the MBR with that obtained in the treatment plant with conventional activated sludge treatment and secondary settling, as well as comparing different membrane technologies.

***European context and emerging pollutants of concern***

In Europe, the situations in which an MBR currently becomes competitive with a conventional activated sludge plant are those in which the reuse of the effluent is considered, as well as for cases in which the land to be occupied plays an important role and/or the watercourse to be discharged is a sensitive area with strict discharge parameters. In this sense, in recent years, the implementation of MBR technology in Spain has enormously increased [18].

Although there is no legislation concerning the concentration of most of the micropollutants present in wastewater, the impact on the indirect reuse of secondary effluents for drinking water (e.g., for aquifer recharge) of emerging pollutants that would have gone unnoticed a few years ago is a matter of debate in recent years. In addition to their potential impact on water reuse, it is generally known that their uncontrolled discharge is detrimental to aquatic life [19,20]. At this point, it should be remembered that the aim of legislation and the ethical responsibility of the bodies responsible for water treatment and purification must be to ensure that all hazardous substances are eliminated from the effluent or that concentrations are reduced to values compatible with the natural environment.

The study of emerging pollutants in wastewater and their treatment and removal from wastewater has recently received a great deal of attention, especially due to their ubiquitous presence in all types of water and their potential impact on the environment. Emerging pollutants are a type of organic pollutant that is introduced into the environment, mainly the terrestrial and marine aquatic environment, in large quantities. These include pharmaceutical residues and drugs, hormones, detergents, phytosanitary products, personal hygiene products (sunscreens, fragrances), stimulants such as caffeine, etc. Recently, different administrations are beginning to limit the presence of some of them, although the effects caused by many of them or their abundance in the environment are largely unknown. Thus, the Directive [6] already includes a table identifying a series of emerging pollutants as priority substances and even establishes emission limits for some of them.

At present, studies on emerging pollutants in WWTPs focus on evaluating the performance of the different stages of treatment processes using activated sludge in the elimination of the most common emerging pollutants, studying the different stages to check their presence and concentration in each one of them [21] and on the evaluation and search for treatments for the elimination of emerging pollutants in WWTPs [22].

The quantity and type of pollutants are highly dependent on the region, so a study in Spain, such as the one proposed here, is necessary to evaluate the effectiveness of the treatment technologies installed for the elimination of these types of pollutants and the possibility of implementation in other WWTPs with reuse objectives.

Studies on the removal of emerging pollutants with membrane technology (MBR) developed in urban WWTPs are scarce, but they show good results in terms of obtaining a higher removal performance of these compounds compared with WWTPs with conventional biological treatment [20]. The cell residence time (CRT) of the MBR in these studies was always significantly higher than those used in conventional technologies, so it can be assumed that the increase in removal was due to an increase in cell residence time, with a consequent higher removal of the hardly biodegradable matter. In the absence of biomass settling problems, MBRs can operate with higher cell residence times, which leads to higher removal efficiencies of biodegradable and hardly biodegradable organic matter. As pointed out by Bouju et al. [23,24], when comparing the degradation of micropollutants in MBR and conventional processes under similar conditions, MBR treatment did not improve the removal of those compounds that conventional treatment already removed at a high rate, nor did it improve the removal of recalcitrant compounds that were poorly removed by conventional treatment. However, there seems to be a slight improvement in the removal of those compounds of medium removal by MBR.

The results obtained in terms of micropollutant removal from an MBR plant with a flat membrane and a WWTP with hollow fiber membranes are then tested in comparison with conventional activated sludge treatment, and the possibility of reusing the effluent according to current regulations [7] will be analyzed.

## 2. Materials and Methods

### 2.1. Analysis of the Facilities under Study

**Figure 1:**
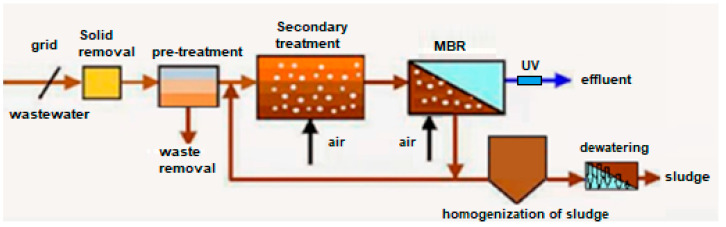
General purification scheme with aerobic biological reactor and membrane biological reactor WWTP A and WWTP B [15].

Two WWTPs located in the Levante area of Spain were selected with the same treatment process diagram but with different types of membrane technology in the MBR system. Figure 1 shows the flow diagram of the two WWTPs.

**Figure 2:**
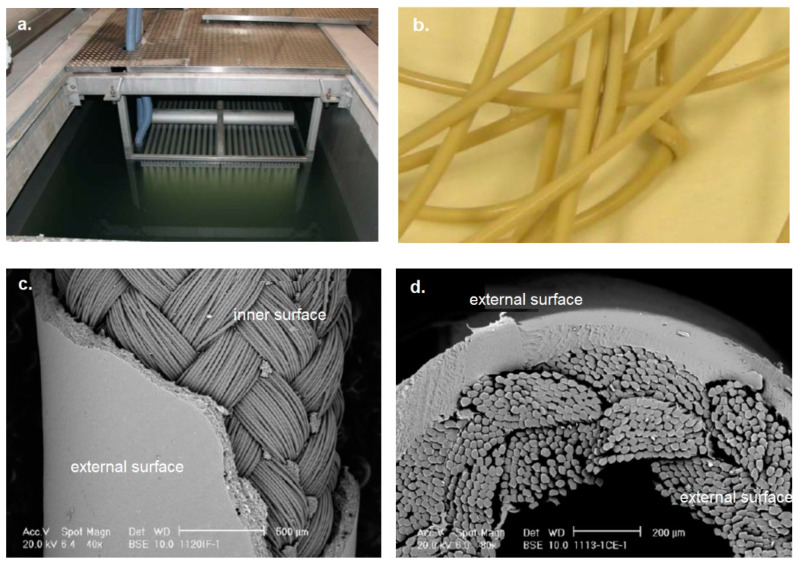
Detail of hollow fiber membrane: (**a**) frame with hollow membrane modules, (**b**) hollow membrane filament, (**c**) lateral image of the inner and external surface of a hollow fiber, and (**d**) frontal image of the inner and external surface of a hollow fiber. WWTP B was designed to treat a flow of 9000 m^3^/day, with the aim of serving a population equivalent to 36,000 equivalent inhabitants. This WWTP has a biological treatment of activated sludge and an MBR system, using Kubota ES200 flat membranes made of chlorinated polyethylene and with a nominal pore size of 0.4 microns maximum (average of 0.2 microns). The ultrafiltration process has 5 treatment lines, consisting of 20 modules of 200 membranes per module. Figure 3a–d show the details of a flat membrane.

WWTP A was designed to treat a flow of 20,000 m^3^/day of wastewater, with the objective of serving a population equivalent to 130,000 inhabitants. The plant incorporates a biological degradation process using activated sludge, which was completed with an ultrafiltration membrane treatment system consisting of Zenon^®^ brand non-ionic and hydrophilic polyvinyl difluoride (PVDF) hollow fiber modules (1516 m^2^ of membrane surface area installed), which provides an effluent of excellent quality without the need for subsequent disinfection of the water, with a significant reduction in the size of the WWTP. The total number of modules: 1536, with a nominal pore diameter of 0.04 μm. Figure 2a–d show the details of the hollow fiber membrane technology installed in the MBR system of WWTP A.

Both plants have a design treatment capacity far in excess of the flow they currently receive, but in both plants, the suspended solids concentration in the mixed liquor in the MBR is similar to the suspended solids concentration in a conventional biological treatment system with conventional settling.

### 2.2. Indicators of Emerging Pollutants

The following 11 compounds were used as model emerging contaminants for the studies conducted: ibuprofen, diclofenac, erythromycin, oxytetracycline, sulfadiazine, carbamazepine, 17-alpha-ethinylestradiol (EE2), triclosan, glyphosate, and imidacloprid, frequently used by the population as antipyretics, analgesics, anti-inflammatories, antibiotics, anticonvulsant drugs, antidepressants, antiseptics, hormones, herbicides, insecticides, etc. The analysis of any additional emerging contaminants and/or their by-products was not ruled out, as this will allow a more robust assessment of the removal capacity of emerging compounds in the secondary and tertiary treatment line.

The proposed minimum indicators have a wide structural and chemical diversity. Therefore, no single analytical method is sufficient to analyze all of them and several analytical methods have to be used, as shown in Table 1.

The proposed minimum indicators have a wide structural and chemical diversity. As a consequence, no single analytical method is sufficient to analyze all of them and several analytical methods have to be used as shown in Table 1.

The analysis of micropollutants was complemented by an analysis of the general chemistry of the samples including ionic composition (ammonium, nitrate, nitrite, phosphate, bromide, chloride, sulfate, sodium, potassium, magnesium, and calcium), basic characterization of dissolved organic matter (ultraviolet and visible absorption spectra, and total dissolved organic carbon), inorganic carbon (alkalinity), pH, conductivity, and turbidity.

### 2.3. Strategy for the Analysis of Pollutants of Emerging Concern

The wide variety of CECs we are confronted with, their different physicochemical properties (molecular structure, pKa, etc.), as well as the complexity of the matrices, complicate the development of a “universal” methodology for the determination of CECs in environmental samples (wastewater, sludge, etc.). Therefore, the selection and optimization of the analytical procedure will be a key factor in the success of the analysis, influencing the reliability, precision, and reproducibility, hence the results obtained.

Although the current trend is the development of rapid, sensitive, and generic methods that allow the extraction and analysis of as many compounds as possible at relatively low costs, the study of CECs requires very specific analytical techniques with highly specialized instrumentation that is not available in any conventional WWTP laboratory. Therefore, a laboratory with extensive experience in the analysis of emerging contaminants in liquid and solid samples, and which has developed multi-compound methods that allow the analysis of a large number of compounds at a similar effort, was used.

First of all, the analytical methods will have to be fine-tuned and adapted to the sample matrix and the concentration range of the pollutants under consideration.

The determination of emerging pollutants is carried out by gas chromatography coupled to a mass spectrometry detector (GCMS). 

**Figure 4:**
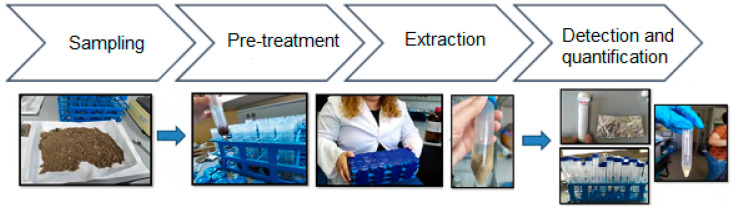
Key steps in the analytical process of CECs extraction in real samples.

The samples cannot be injected directly and pre-treatment is necessary to obtain the species of interest under optimal conditions for their measurement. To this end, the water samples will be subjected to a pre-treatment [31], an extraction step [32,33,34,35], and subsequent clean-up of the extracts and, finally, the detection and quantification of the CECs in the GCMS (see Figure 4) [36,37,38]. 

The sample injected into the GCMS will determine the concentration of contaminants by performing a calibration with standard compounds. The planning of each of these steps is very important, as a poorly defined strategy could lead to a wrong answer.

### 2.4. Work Plan

The project will be carried out over a period of one year at three main points:

– Raw water at the inlet of the treatment plant (M1);
– After biological treatment (M2);
– Treated water leaving the MBR (M3);
– Samples of the accumulated sludge (M4) are also taken.

**Figure 5:**
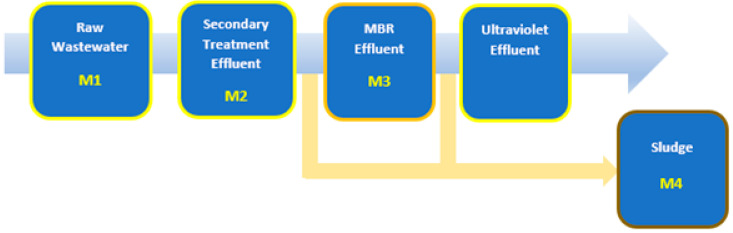
Control and sampling points.

Figure 5 illustrates the sampling points in a generalized scheme of the two sampled WWTPs.

The type of sample can be:

– Single sample: a discrete sample taken at a given time and place. The main disadvantage of this type of sampling is the uncertainty as to the choice of the optimal time to take the sample, due to the variations that may occur during the day.

The main disadvantage of this type of sampling is uncertainty as to the optimum time to take the sample, due to the variations that may occur during the day.

– Composite or integrated sampling: samples collected at the same point at different times (over 24 h). This type of sample takes into account the usual variability of a WWTP, since discharges of different characteristics may occur within a day.

The work is divided into two phases:

Phase I: initial evaluation of the variability and presence of micropollutants in WWTP A and WWTP B [39,40].

In this phase, two objectives are established:

1. Initial assessment of the variability and prevalence of micropollutants in the wastewater entering the WWTPs under study (M1).
2. To assess the need for sampling at point M2 in order to decide on consecutive sampling.

All liquid samples will be collected in 24 h with automatic samplers.

In the initial sampling, integrated samples are taken over 24 h at points M1, M2, and M3 on three consecutive days, resulting in a total of 9 integrated samples.

Samples are also taken from the accumulated sludge (M4) over these days, generating an integrated sample for the three days. These samples are analyzed with the aforementioned analytical methods: drugs, watch list, and glyphosate, following their corresponding pre-treatment.

As described above, the general chemistry of the samples is also analyzed (ionic composition including nutrients, characterization of dissolved organic carbon, etc.).

Phase II: periodic sampling of microcontaminant occurrence in WWTP A and WWTP B.

The objective of this phase is to evaluate the variability and prevalence of pollutants in the raw water input and output water of the WWTPs over 12 months.

In 12 months, integrated samples were taken monthly over 24 h at points M1, M2, and M3 on three consecutive days, resulting in a total of 108 integrated samples. All samples were analyzed for general chemistry in an accredited external laboratory as described above. From 72 samples taken at points M1 and M3, the micropollutants were analyzed with the above-mentioned methods: drugs, watch list, and glyphosate following their corresponding pre-treatment. The M2 samples were kept frozen in order to be able to refer to them in case of doubt in the interpretation of the results.

In each sampling of this phase, an integrated sample of the sludge line (M4) was also taken over 3 days. Its analysis was decided on the basis of the results obtained in phase 1, as most of the hydrophilic contaminants are not found in the sludge.

## 3. Results

The results show the average concentrations of the compounds detected in the influent and effluent of WWTP A (Table 2) and WWTP B (Table 3).

The performances obtained in the elimination of the different selected emerging pollutants (Table 1) are expressed in percentage of reduction or retention coefficient, according to the formula:

(1) $$R\;\left(\%\right)=\frac{\left(\mathrm{Cf}-\mathrm{Cp}\right)}{\mathrm{Cf}}\times 100$$

where Cf is the concentration of pollutants in the raw wastewater and Cp is the concentration of pollutants at the outlet of the corresponding treatment (secondary treatment, MBR hollow fiber, and MBR flat fiber).

As a starting point, we can verify that the water permeated by both membrane technologies complies with the regulations according to the reuse law [6] for all urban, agricultural, industrial, and irrigation uses, which was to be expected if we consider that the average pore size of the membranes is significantly higher than the average size of the microorganisms studied. As can be seen in Table 4.

The analysis of the general chemistry, as a measure to verify the correct functioning of the WWTPs, shows high removal efficiencies for organic compounds in both membrane processes [41,42].

**Figure 6:**
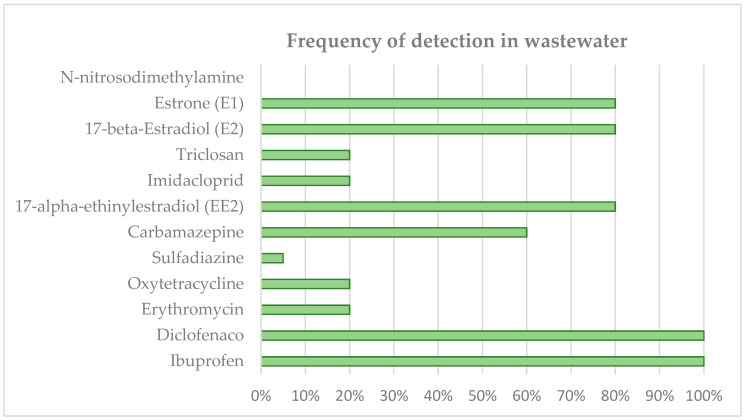
Frequency of detection of emerging pollutants at the inlet of WWTP A.

**Figure 7:**
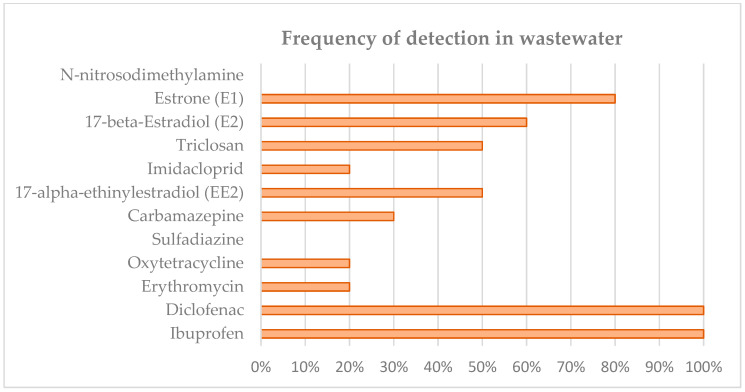
Frequency of detection of emerging pollutants at the inlet of WWTP B.

If we focus on the micropollutant monitoring campaign carried out at WWTP A and WWTP B, we detected the presence of a very large number of emerging pollutants in the water entering the WWTPs. The most common drugs are pharmaceuticals (anti-inflammatory, analgesic, anti-epileptic and antibiotic drugs), disinfectants, herbicides, etc. Figure 6 and Figure 7 show the average results obtained in the analysis of all the raw water samples for the different pollutants. The pharmaceuticals found in the influent and effluent are in line with those found in the literature for other WWTPs [43,44].

A number of compounds are found more frequently (E1, E2, EE2, carbamazepine, diclofenac, and ibuprofen). And there are a number of compounds that do not occur or occur sporadically and in low concentrations: N-nitrosodimethylamine, imidacloprid, sulfadiazine, oxytetracycline, erythromycin, triclosan, etc.).

It should be noted that pollutants are also indicators of the origin of the influents to the WWTPs, appearing depending on the discharges received in the sewage networks. Thus, compounds such as ibuprofen, diclofenac, E1, E2, carbamazepine, etc., are abundant in all influents, indicators of their wide use in the population, some of them in the geriatric population.

The results of the average concentrations of the selected emerging pollutants detected in the influent and effluent of WWTP A are shown in Table 5 and for WWTP B in Table 6.

**Figure 8:**
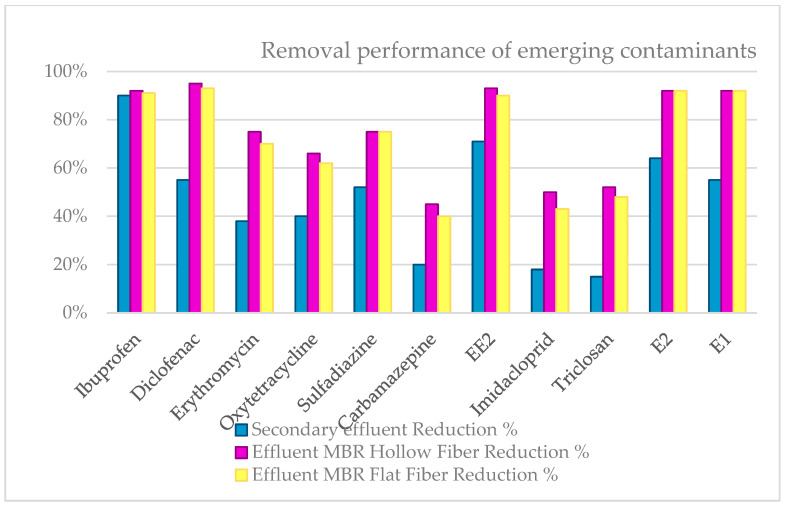
Comparison of removal efficiencies of emerging pollutants in secondary treatment WWTP, WWTP A, and WWTP B.

From the data obtained and shown in Table 5 and Table 6, the removal efficiencies of the selected emerging pollutants are calculated for each of the treatment stages (Figure 8).

If the compounds are grouped by families, it can be concluded that the removal performance after secondary treatment had the following distribution:

– Very low (10–20%): antiepileptic, insecticide, and antiseptic.
– Low (30–40%): antibiotics.
– Medium (50–60%): anti-inflammatories, antibiotics, and hormonal.
– High (60–70%): contraceptives and hormonal.
– Very high <90: Anti-inflammatories.

After tertiary treatment by MBR with flat ultrafiltration membranes and hollow fiber, the following removal efficiencies was achieved:

– Medium (40–60%): antiepileptic, antiseptic, and insecticide.
– High (60–80%): antibiotics.
– Very high <90%: anti-inflammatory, hormonal, and contraceptives.

In general, the same compounds are seen at the inlet as at the outlet. However, the frequency of detection and concentration decreases very significantly after treatment at the WWTP. Some compounds are always detected (diclofenac, carbamazepine, etc.), while others, such as ibuprofen, are mineralized or absorbed on the activated sludge, and the removal performance is quite good in all processes. The significant difference detected for ibuprofen can be explained by the average amount detected. The other compounds were found in a much lower concentration.

The yields obtained with hollow membrane technology are slightly higher than those obtained with flat fiber membrane technology. In both cases, the eliminations were significantly higher than those found with conventional biological treatment. This effect is more pronounced in the case of those compounds that show low removals in conventional water treatment processes, such as the anti-inflammatory diclofenac and the anti-epileptic carbamazepine [20]. The results obtained were expected due to the difference in the pore size of the two membranes.

For pollutants that are difficult to remove using MBR technology with ultrafiltration membranes (erythromycin, oxytetracycline, carbamazepine, etc.), as shown in Figure 8, the ultraviolet (UV) radiation system available in each of the facilities under study must be put into operation to improve the results in terms of the removal percentages achieved. The high quality of the water obtained by the MBR systems means that the ultraviolet radiation technology has very high yields.

On the other hand, only a few compounds (oxytetracycline, carbamazepine, triclosan, and ibuprofen) were detected in the sludge, the compounds with the highest concentration detected. The limit of quantification is 0.10 µg/L, so it is difficult to detect compounds with low concentrations.

In future sampling, the obtained results will be evaluated with standard statistical methods, e.g., trends over time (correlation with seasons and linear time to discover patterns) and correlations with general water chemistry parameters determined, especially with the characteristics and amount of dissolved organic matter following the methodologies proposed by Gerrity et al. (2012) and Lee et al. (2013) [45,46], with the very aim to know and follow the treatment performances.

## 4. Discussion and Conclusions

Membrane bioreactors are a viable and robust alternative for water reuse in Spain. Despite the high variability of the input water and the unforeseen events that may arise in the normal course of operation of these systems, the quality of the output water is not significantly affected and complies at all times with the Reuse Law for all urban, agricultural, industrial, and irrigation uses, as has been proven in the average concentrations obtained in the usual parameters for monitoring treatment in WWTPs.

The study carried out on the effluent shows the presence of pollutants in the sewage network, and although biological treatment manages to reduce the concentration of a large part of them, it requires a more advanced process. With the data obtained, the application of tertiary treatment by membranes manages to substantially reduce a large part of these compounds. During the study, no significant differences were found between the results obtained with flat membrane MBR and hollow fiber MBR operation.

The results showed that some micropollutants were removed fairly well with biological treatment (ibuprofen) and others to a greater extent with MBR technology, the difference can be attributed to the fact that the effluent is filtered through a pore size in the order of 0.2–0.04 μm, avoiding the problems of sludge settling in the decanters with solids leaking into the effluent.

The removal mechanisms for compounds with some biodegradability and decantability could be removed by conventional scrubbing treatment, but for compounds that are found in smaller quantities and in suspension, filtration by low pore sizes can contribute to removal.

In this study, the residence time of the cells of the MBR systems of WWTP A and B was similar to that of a conventional WWTP, so this factor has not contributed substantially to the increase in the removal performance of compounds.

The studies carried out are preliminary, so further study is required. It is also necessary to study in more detail how the operating conditions and the variability of discharges affect the removal of compounds. The removal mechanisms also require further investiation in more detail.

With regard to the results obtained in the removal of micro and macro pollutants for the hollow fiber and the flat membrane, no significant differences were found, with the effluent from both technologies being of high quality. The slight difference obtained between the two membranes can be attributed to the difference in pore size on their surface. Better results are obtained with the hollow fiber membrane technology.

Analyses carried out in two urban wastewater treatment plants with membrane treatment on emerging pollutants of different nature (pharmaceuticals, hormones, disinfectants, pesticides, stimulants, and drugs) show that, although a large part is removed in secondary treatment and MBR systems, others remain in the final effluent, ending up in the environment.

For the more complicated cases of removal of emerging contaminants in wastewater for agricultural reuse, the key advances and challenges to remove these organic contaminants lead to the combination of technologies: MBR and ozonation, MBR and peracetic acid, and advanced oxidation processes (UV/TiO2, photo-Fenton, etc.).

As mentioned above, the aim of this study was not only to evaluate the removal performance of emerging compounds with membrane technology for possible implementation in other facilities that require it, but also to serve as a basis for establishing protocols for the control of pollutants and risk assessment, both for health and for the environment.

We, therefore, consider that the identification of environmental pathways of pharmaceuticals and personal care products and their metabolites is a key source of knowledge to understand the different sources of contamination and the actual burden of these contaminants in the environment. This represents a breakthrough in their treatment. Appropriate purification treatments at the source, together with the responsible use of the products by the population, would moderate their arrival at WWTPs, reducing their discharge into the environment and avoiding more costly and environmentally less sustainable final treatments, which may end up being necessary in the face of future legal regulations [44].

Given the importance of the appearance of emerging pollutants in the environment, which are residues that are difficult to treat, highly persistent, and with unpredictable consequences for humans, animals, and the natural environment, it is essential, rather than monitoring their presence and concentration as proposed by many authors [47,48], to take the following measures and carry out emergency actions to reduce them at the source:

– Carrying out an environmental assessment of pharmaceutical products before they are marketed and substituting those that are more harmful to the environment.
– Apply the Registration, Evaluation, Authorisation, and Restriction of Chemicals (REACH) regulation to these compounds [49].
– Promote education and good practices among producers, distributors, and consumers for the rational use of these substances.
– Provide specific treatment at the main emission sources such as hospitals, health centers, nursing homes, the pharmaceutical industry, etc.

In general, the same compounds are seen at the inlet as at the outlet. However, the frequency of detection and concentration decreases very significantly after treatment at the WWTP. Some compounds are always detected (diclofenac, carbamazepine, etc.), while others, such as ibuprofen, are mineralized or absorbed on the activated sludge, and the removal performance is quite good in all processes. The significant difference detected for ibuprofen can be explained by the average amount detected. The other compounds were found in a much lower concentration.

## Figures and Tables

**Figure 3:**
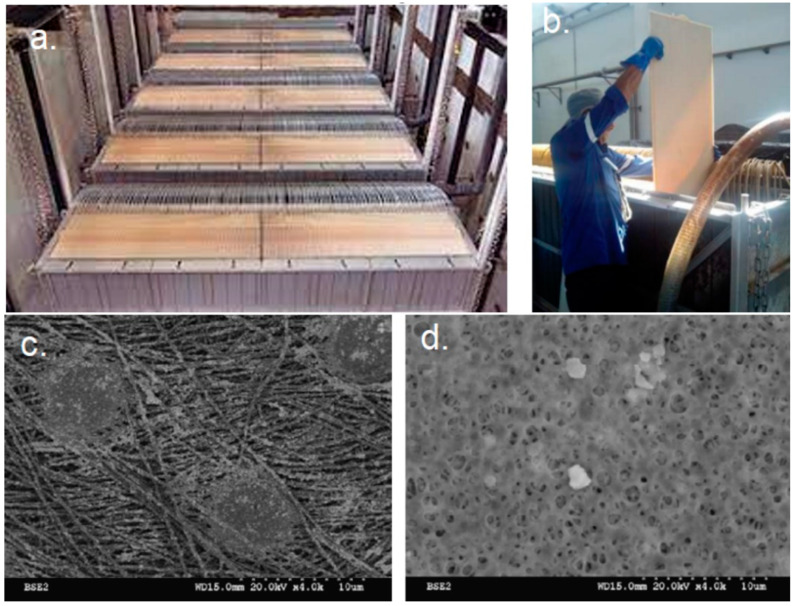
Detail of flat fiber membrane [25]: (**a**) frame with flat membrane modules, (**b**) flat membrane sheet, (**c**) image of the inner surface of a flat membrane, and (**d**) image of the external surface of a flat membrane.

**Table 1:** Summary of compounds analyzed and analytical methods used.

Family	Analytical Methods	Indicators Proposed	Group
Pharmaceuticals	Solid phase extraction (SPE) in cartridge, liquid chromatography-mass spectrometry (HPLC-MS) analysis	Ibuprofen	Anti-inflammatory
		Diclofenac	Anti-inflammatory
		Erythromycin	Antibiotic
		Oxytetracycline	Antibiotic
		Sulfadiazine	Antibiotic
		Carbamazepine	Antiepileptic
Watch list	On-line solid phase extraction (SPE) on the same HPLC-MS equipment	17-alpha-Estradiol (EE2)	Hormone
		Erythromycin	Antibiotic
		Diclofenac	Anti-inflammatory
		Imidacloprid	Insecticide
		Triclosan	Antiseptic
		17-beta-Estradiol (E2)	Contraceptive
		Estrone (E1) [26]	Hormone
Glyphosate [27,28,29]	Glyphosate derivatization, SPE extraction, and HPLC-MS	Glyphosate	Herbicide
Nitrosamines [30]	SPE extraction, gas chromatography-mass spectrometry (GC-MS) analysis	N-nitrosodimethylamine	Industrial products

**Table 2:** Average concentrations of the usual parameters for treatment monitoring at WWTP A.

Control Parameters	Analytical Method	Wastewater	Secondary Effluent	Effluent MBR Hollow Fiber
Suspended solids (mg/L)	Gravimetric analysis	196	14	3.7
Biological oxygen demand (mg/L)	Incubation and electrometry	177	17.7	7.5
Chemical oxygen Demand (mg/L)	Photometric analysis	392	23.5	23.8
Total nitrogen (mg/L)	Photometric analysis	60.2	6	4.2
P-total phosphorus (mg/L)	Photometric analysis	5.7	2.9	2.3
Escherichia coli (ufc/100 mL)	Membrane filtration counting	8.5 × 10^5^	1.1 × 10^5^	<10
Clostridium spores (ufc/100 mL)	Membrane filtration counting	1.5 × 10^5^	5.25 × 10^4^	4400
Helminths (number of eggs/L)	Modified Bailinger method	5 × 10^5^	1.5 × 10^5^	<1

**Table 3:** Average concentrations of the usual parameters for treatment monitoring at WWTP B.

Control Parameters	Analytical Method	Wastewater	Secondary Effluent	Effluent MBR Flat Fiber
Suspended solids (mg/L)	Gravimetric analysis	206	13.7	4.5
Biological oxygen demand (mg/L)	Incubation and electrometry	503	50.3	18.5
Chemical oxygen demand (mg/L)	Photometric analysis	651	39	46
Total nitrogen (mg/L)	Photometric analysis	72.3	7.2	5
P-total phosphorus (mg/L)	Photometric analysis	15.8	7.9	5.5
Escherichia coli (ufc/100 mL)	Membrane filtration counting	16.5 × 10^5^	21.5 × 10^4^	<10
Clostridium spores (ufc/100 mL)	Membrane filtration counting	18 × 10^4^	6.3 × 10^4^	2650
Helminths (number of eggs/L)	Modified Bailinger method	30.5 × 10^5^	91.5 × 10^4^	<1

**Table 4:** Average pollutant removal efficiencies with different treatment technologies.

Control Parameters	Secondary Effluent Reduction %	Effluent MBR Hollow Fiber Reduction %	Effluent MBR Flat Fiber Reduction %
Suspended solids	93	98	98
Biological oxygen demand	90	96	96
Chemical oxygen demand	94	94	93
Total nitrogen	90	93	93
P-total phosphorus	50	60	65
Escherichia coli	87	99	99
Clostridium spores	65	99	99
Helminths	70	99	99

**Table 5:** Average concentrations of the emerging pollutants selected as a model for monitoring at WWTP A.

Control Parameters	Wastewater	Secondary Effluent	Effluent MBR Hollow Fiber	Sludge
Ibuprofen (µg/L)	14.02	1.40	1.12	0
Diclofenac (µg/L)	0.85	0.38	0.04	0
Erythromycin (µg/L)	0.7	0.43	0.18	0
Oxytetracycline (µg/L)	1.6	0.96	0.54	0.21
Sulfadiazine (µg/L)	0.09	0.04	0.02	0
Carbamazepine (µg/L)	0.3	0.24	0.17	0
EE2 (µg/L)	1.7	0.49	0.12	0
Imidacloprid (µg/L)	0.52	0.43	0.26	0
Triclosan (µg/L)	1.9	1.62	0.91	0.32
E2 (µg/L)	1.54	0.55	0.12	0
E1 (µg/L)	2.16	0.97	0.17	0
N-nitrosodimethylamine (µg/L)	0	0	0	0

**Table 6:** Average concentrations of the emerging pollutants selected as a model for monitoring at WWTP B.

Control Parameters	Wastewater	Secondary Effluent	Effluent MBR Hollow Fiber	Sludge
Ibuprofen (µg/L)	17.4	1.74	1.57	0.25
Diclofenac (µg/L)	0.52	0.23	0.04	0
Erythromycin (µg/L)	1	0.62	0.30	0
Oxytetracycline (µg/L)	0.9	0.54	0.34	0
Sulfadiazine (µg/L)	0	0.00	0.00	0
Carbamazepine (µg/L)	0.71	0.57	0.43	0.12
EE2 (µg/L)	2.5	0.73	0.25	0
Imidacloprid (µg/L)	0.43	0.35	0.25	0
Triclosan (µg/L)	1.34	1.14	0.70	0.43
E2 (µg/L)	1.7	0.61	0.14	0
E1 (µg/L)	1.35	0.61	0.11	0
N-nitrosodimethylamine (µg/L)	0	0	0	0

## Data Availability

The authors choose to exclude this statement.
